# Multimodal imaging features of adult-onset Coats disease: A case series

**DOI:** 10.1097/MD.0000000000051065

**Published:** 2026-10-09

**Authors:** Guangwei Yu, Fanchao Meng, Feng Chen, Haohao Lu, Yanhui Han

**Affiliations:** a Department of Ophthalmology, Jinan Mingshui Eye Hospital, Jinan, Shandong, China.

**Keywords:** adult-onset, Coats disease, multimodal imaging

## Abstract

The imaging manifestations of adult-onset Coats disease differ from those in children. This study explored the multimodal imaging features of adult-onset Coats disease. This study included patients diagnosed with adult-onset Coats disease (>18 years) from December 2021 to December 2024 at Jinan Mingshui Eye Hospital. Data on best corrected visual acuity (BCVA), computerized optometry, intraocular pressure (IOP), slit lamp microscope, indirect ophthalmoscope, ocular B-ultrasound, ultra-wide field fundus imaging (UWFI), ultra-wide field fluorescein fundus angiography (UW-FFA), and optical coherence tomography (OCT) were collected. The study included fifteen patients (16 eyes). The mean subject age was 51 ± 11.8 years, with 12 males (13 eyes) and 3 females (3 eyes). Fourteen patients showed painless decline in monocular vision, and 1 patient had no symptoms. The affected eyes had a median logMAR BCVA of 1.10 (range 0.00–2.00). Ocular B-ultrasonography showed no massive vitreous opacities in all patients; 2 cases were reported with localized exudative retinal detachment, and 3 cases showed localized solid elevations adjacent to the ocular wall. UWFI examination showed yellow-white hard exudation in all patients, varying in scope and degree. Some arteriole branches showed a “lightbulb” appearance, and small bleeding was interspersed around them. There were 13 cases with exudation involving the macula. Fundus lesions were consistent with typical Coats disease exudation in 6 cases (40.0%), Leber miliary aneurysm (LMA) in 4 cases (26.7%), and macular telangiectasia type 1 (MacTel 1) in 5 cases (33.3%). UW-FFA examination showed convoluted and dilated small vessels in the fundus lesion, and the vascular wall showed cystic, fusiform, or bead-like neoplasia changes. No perfusion areas of varying degrees could be seen in the peripheral area in the early stage of all patients. In the late stage, abnormal angiofluorescein leakage and staining were observed. FFA showed that the lesions involved the macula in 14 cases, and fundus abnormalities in fellow eyes were found in 8 patients (53.3%). OCT examination showed different degrees of macular edema with hyperreflective mass in 14 cases and epiretinal membranes in 5 cases. Adult-onset Coats disease is rare in the clinic, and the range and types of fundus lesions are diverse. More than half of the patients have abnormalities in FFA in the fellow eye, and a detailed examination should be carried out in combination with multiple imaging modes, especially UW-FFA.

## 1. Introduction

Coats disease is an idiopathic retinal vascular disorder first described by George Coats in 1908, characterized by retinal telangiectasia, microaneurysm formation, and intraretinal and subretinal lipid exudation. Leber miliary aneurysms were once considered a distinct entity, but were later confirmed by Reese to represent an early manifestation of Coats disease. Previous studies have suggested that Coats disease predominantly affects male infants, children, or adolescents. However, in recent years, Rishi et al reported that among 646 cases of Coats disease, 7.0% were adult-onset Coats disease.^[1-3]^ The clinical presentation of adult-onset Coats disease resembles that of the childhood form, but with the following features: slower progression, more localized lipid deposition, and concurrent involvement of the peripheral and juxtamacular regions. Compared with children, adult patients present at an earlier disease stage, with less macular involvement and better baseline visual acuity. The childhood form more frequently exhibits extensive exudation and more macular optical coherence tomography (OCT) abnormalities. The adult form tends to have a more limited disease extent, a more benign natural course, and better treatment outcomes.^[3]^ In the literature,^[4-6]^ typical adult-onset Coats disease, Leber’s miliary aneurysm (LMA), and idiopathic Macular telangiectasia type 1 (MacTel 1) are often grouped under the category of Coats disease. Ultra-wide field fundus imaging (UWFI) identifies more retinal pathology than conventional fundus imaging and guides precise laser photocoagulation therapy. Despite the convenience of UWFI in the assessment of Coats disease, comprehensive reports on the multimodal imaging characteristics of adult-onset Coats disease in the Chinese population are still scarce. This study retrospectively analyzed the clinical data of 15 patients diagnosed with adult-onset Coats disease based on clinical examination, focusing on describing their imaging characteristics. The results are reported as follows.

## 2. Materials and methods

### 2.1. Study design and participants

This retrospective study included patients diagnosed with adult-onset Coats disease (>18 years) from December 2021 to December 2024 at Jinan Mingshui Eye Hospital. The inclusion criteria were: age of onset >18 years; UWFI and ultra-widefield fluorescein fundus angiography (UW-FFA) showing abnormal dilation of retinal capillaries and microvessels, accompanied by extensive lipid exudation, with or without exudative retinal detachment; early-phase FFA revealing tortuous and dilated small retinal vessels with sac-like, spindle-shaped, or beaded aneurysmal changes, hemorrhage and exudates blocking fluorescence, and faint fluorescence blockage beneath large and medium retinal vessels in cases of extensive exudation, peripheral non-perfusion areas also observed; late-phase FFA showing fluorescein leakage and staining from abnormal vessels. The exclusion criteria were: age of onset <18 years or Coats disease diagnosed before the age of 18; presence of any other vascular or inflammatory retinal diseases that may affect clinical judgment, such as retinal vein occlusion (RVO), diabetic retinopathy (DR), posterior uveitis, retinal vasculitis, acute retinal necrosis, retinitis pigmentosa (RP), etc; coexistence with other genetic metabolic diseases or syndromes, such as facioscapulohumeral muscular dystrophy (FSHD), Parry-Romberg syndrome (PRS), etc; history of any intraocular surgery; presence of severe refractive media opacities such as cataract or corneal opacity that obstruct fundus examination; and intraocular tumors (e.g., retinoblastoma, choroidal melanoma, Von Hippel–Lindau disease) based on B-scan ultrasonography and FFA. This study was approved by the ethics committee of Jinan Mingshui Eye Hospital, and informed consent was waived due to its retrospective nature.

### 2.2. Data collection and definition

Data were obtained from medical records, including age and gender. All patients underwent examinations including best-corrected visual acuity (BCVA), automated refraction, intraocular pressure (IOP) measurement, slit-lamp microscopy, indirect ophthalmoscopy, ocular B‑scan ultrasonography, UWFI, UW‑FFA, and OCT. UWFI and UW‑FFA were performed using the Optos200Tx laser scanning ophthalmoscope (Optos, UK); OCT examinations were conducted using the Spectralis HRA OCT (Heidelberg, Germany), DRI OCT Triton (TOPCON, Japan), and OCT VG200 (Intalight, China). All imaging procedures were carried out by skilled physicians, and the results were independently reviewed and analyzed by 2 experienced specialists. After definitive diagnosis, the treatments administered were as follows: combined intravitreal injection of anti‑VEGF agents and retinal laser photocoagulation in 7 eyes, retinal laser photocoagulation alone in 3 eyes, intravitreal anti‑VEGF injection alone in 2 eyes, combined intravitreal anti‑VEGF injection and vitrectomy in 1 eye, cryotherapy in 1 eye, while 2 eyes received no treatment due to patient refusal. A retrospective analysis was performed on the patients’ general clinical data and multimodal imaging features.

### 2.3. Statistical analysis

SPSS 21.0 (SPSS Inc., Chicago) was used for statistical analysis. Continuous variables were summarized as mean ± standard deviation or median (range) as appropriate, and categorical data were presented as n (%). As this was a retrospective descriptive study, only descriptive statistics were applied, and no inferential or comparative analyses were performed.

## 3. Results

### 3.1. Basic characteristics

A total of 15 patients (16 eyes) were included for analysis. Among the patients, there were 3 females (3 eyes) and 12 males (13 eyes). The mean age was 51 ± 11.8 years, with a range from 25 to 67 years. Chief complaints included decreased vision in 14 cases, while 1 case was asymptomatic. Hypertension was present in 6 cases, all medically well-controlled. The affected eyes had a median logMAR BCVA of 1.10 (range 0.00–2.00) and a mean IOP of 15.6 ± 3.1 mm Hg. Among the 15 patients, 11 eyes were left eyes and 5 were right eyes. According to the Shields^[7]^ classification for Coats disease, stages included 2A in 2 eyes (12.5%), 2B in 12 eyes (75.0%), and 3A in 2 eyes (12.5%). General patient data and important multimodal imaging feature presentations are detailed in Table 1.

**Table 1:** General patient data and important multimodal imaging features.

Serial no.	Serial no.	Age	Gender	Eye involved		BCVA		Spherical	Macular involvement	Macular edema	Macular involvement	Abnormalities in peripheral
(by total)	(by exudates)				OD		OS	equivalent (D)	of exudate	on OCT	on FFA	FFA of the fellow eye
1	C1	33	Female	OS	1.0		0.5	−0.50	No	Yes	Yes	2–3
2	C2	53	Male	OS	0.8		0.08	−0.75	Yes	Yes	Yes	No
3	C3	25	Female	OS	1.0		1.0	−3.50	No	No	No	1–2–3
4	C4	37	Male	OU	0.4		1.0	−6.25	Yes	Yes	Yes	Coats lesion
5	C5	52	Female	OD	0.04		0.6	−3.00	Yes	Yes	Yes	No
6	C6	55	Male	OD	0.02		0.15	−15.75	Yes	Yes	Yes	NO
7	L1	56	Male	OD	0.04		1.2	+1.00	Yes	Yes	Yes	1–2–3–4
8	L2	58	Male	OS	1.0		0.02	+2.25	Yes	Yes	Yes	2–4
9	L3	67	Male	OS	0.8		0.04	−0.25	Yes	Yes	Yes	1–2
10	L4	67	Male	OS	0.8		0.01	+1.00	Yes	Yes	Yes	1–2–3–4
11	M1	45	Male	OS	1.2		0.02	+0.50	Yes	Yes	Yes	No
12	M2	48	Male	OS	1.0		0.12	0	Yes	Yes	Yes	1–2–3–4
13	M3	53	Male	OS	1.0		0.4	−0.75	Yes	Yes	Yes	No
14	M4	57	Male	OD	0.4		1.0	+0.25	Yes	Yes	Yes	No
15	M5	59	Male	OS	1.0		0.3	+0.50	Yes	Yes	Yes	No

BCVA (Snellen decimal). Fundus lesions are consistent with typical Coats disease exudation (labeled C), consistent with Leber’s miliary aneurysm (labeled L), and consistent with Macular telangiectasia type 1 (labeled M). Involvement of the macula is defined as being <500 μm from the foveal center. Abnormalities in the peripheral FFA of the fellow eye include: 1 peripheral small vessel tortuosity, 2 capillary dilation, 3 mild leakage, and 4 microaneurysms.

BCVA = best-corrected visual acuity, OCT = optical coherence tomography, FFA = fluorescein angiography.

### 3.2. Multimodal imaging feature presentations

Ocular B-scan ultrasound examination showed no massive vitreous opacities in all patients. Two cases were reported with localized exudative retinal detachment (case 1, 2), and 3 cases showed localized solid elevations adjacent to the ocular wall (case 4, 5, 6).

UWFI revealed yellowish-white hard exudates in all patients, varying in extent and severity. Some small arterial branch vessels exhibited bulbous dilation resembling a “light bulb” appearance, accompanied by surrounding small hemorrhages. Exudative lesions involved the macula in 13 cases, including 1 male patient with bilateral involvement (case 4) who had extensive yellowish-white exudates affecting the macula in the right eye and peripheral exudates in the left eye. Fundus lesions were consistent with typical Coats disease exudation in 6 cases (40.0%; case C1–C6) (corresponding to Fig. 1A–F), with Leber miliary aneurysms in 4 cases (26.7%; case L1–L4; Fig. 1G, H corresponding to L1, L3), and with type 1 macular telangiectasia in 5 cases (33.3%; case M1–M5; Fig. 1I, J corresponding to M2, M5; Table 1 and Fig. 1).

**Figure 1.**
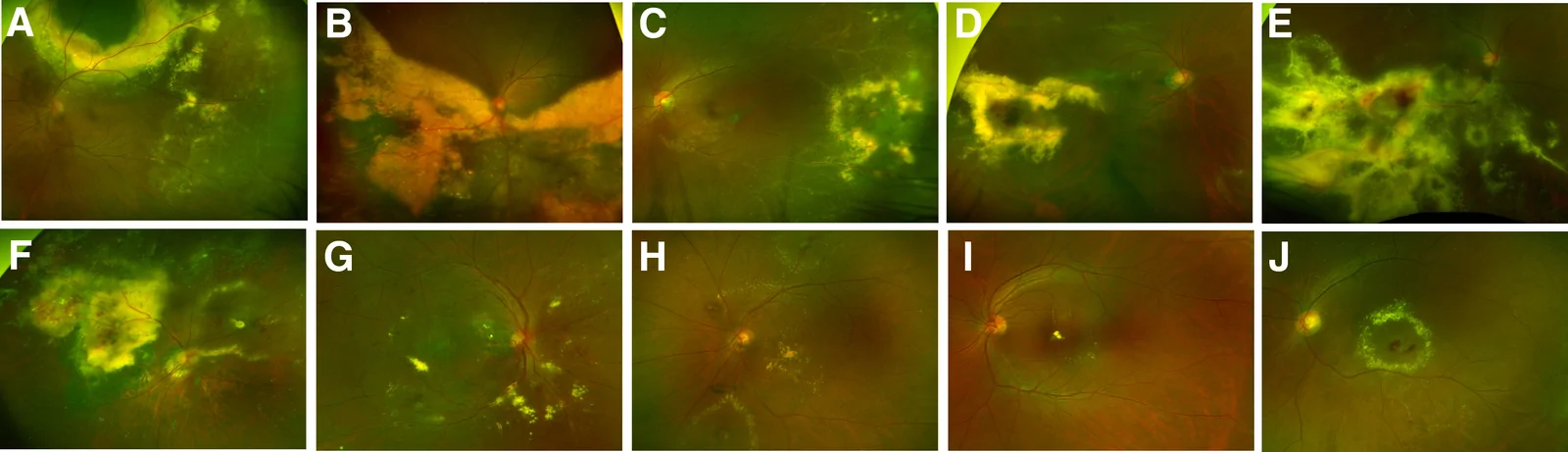
Ultra-widefield fundus imaging (UWFI) findings in adult-onset Coats disease. (A–F) Figures correspond to cases C1–C6 and show typical exudation in adult-onset Coats disease, with large areas of yellow-white retinal exudates. (G, H) Figures correspond to cases L1 and L3 and show Leber miliary aneurysm, with multiple localized aneurysmal dilations along the retinal arteries, mainly in the midperipheral or far-peripheral fundus, accompanied by scattered yellow-white hard exudates, retinal hemorrhages, and macular edema. (I, J) Figures correspond to cases M2 and M5 and show type 1 macular telangiectasia, with yellow-white hard exudates in the macular area, macular edema, and localized hemorrhage. UWFI = ultra-wide field fundus imaging.

UW-FFA demonstrated tortuous and dilated small vessels in the lesion areas during the early phase, with vessel walls showing cystic, fusiform, or beaded aneurysmal changes. Varying degrees of non-perfusion areas were observed in the peripheral retina. Late-phase imaging revealed fluorescein leakage and staining from abnormal vessels. FFA indicated macular involvement in 14 cases (Fig. 2A–C corresponding to C3, L3, M2). Abnormalities in the contralateral fundus were present in 8 patients (53.3%). All cases consistent with LMA (L1–L4) showed varying degrees of abnormalities in the peripheral retina of the contralateral eye on FFA, such as peripheral vascular tortuosity, capillary dilation, mild leakage, and a few microaneurysms (Fig. 2D, E showing temporal peripheral regions of the contralateral eyes in L1 and L3). Cases consistent with MacTel 1 (M1–M5) exhibited not only FFA abnormalities in the macular area but also varying degrees of peripheral retinal abnormalities, including non-perfusion areas, peripheral vascular tortuosity, capillary dilation, mild leakage, and a few microaneurysms (Fig. 2F showing nasal peripheral region in the affected eye of M2; Fig. 2G showing temporal peripheral region in the affected eye of M5; Table 1 and Fig. 2).

**Figure 2.**
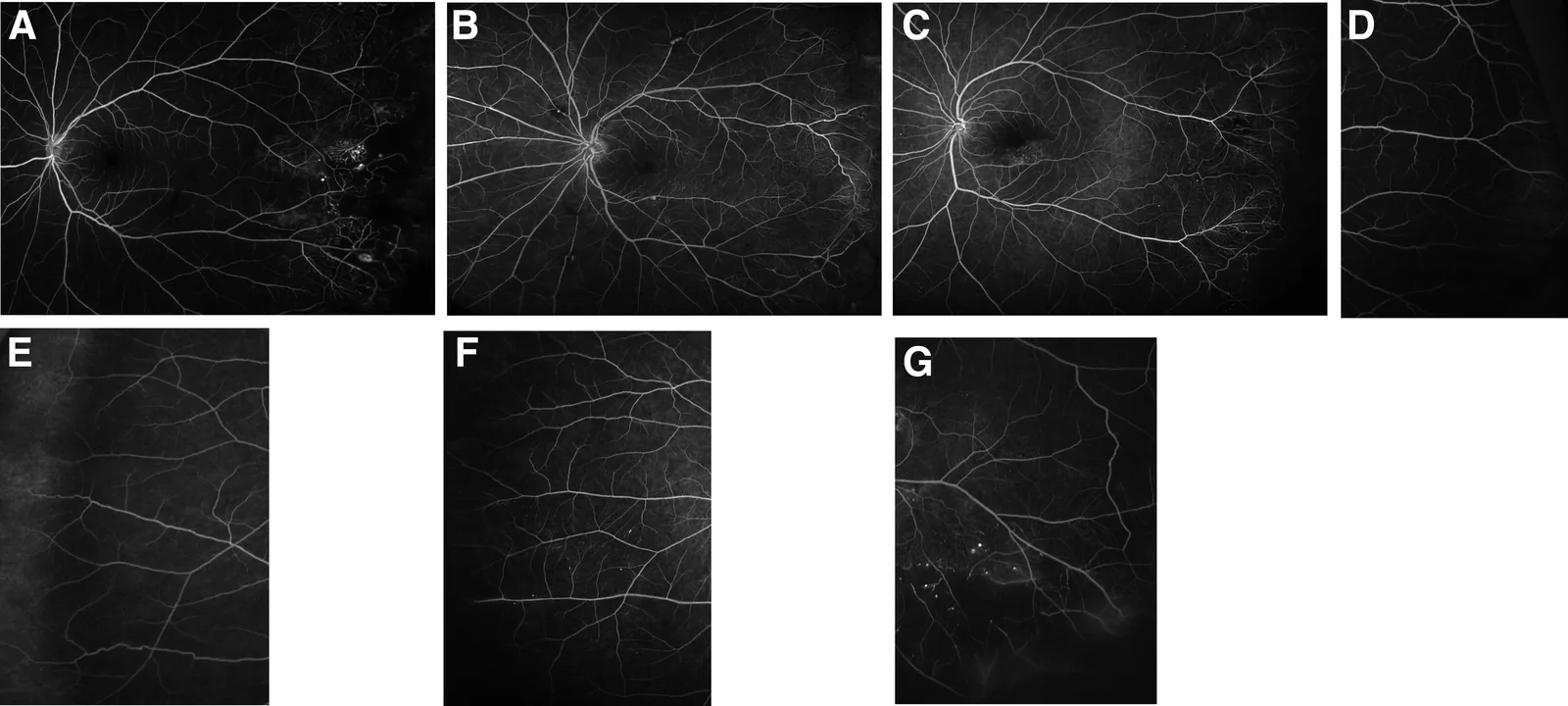
Ultra-widefield fluorescein fundus angiography (UW-FFA) findings in adult-onset Coats disease. (A) Figure corresponds to case C3 (UWFI, Fig. 1C) and shows typical adult-onset Coats disease, with tortuous and dilated small vessels showing saccular or fusiform aneurysmal changes. Peripheral nonperfusion areas and fluorescein leakage are visible. (B) Figure corresponds to case L3 (UWFI, Fig. 1H) and shows Leber miliary aneurysm, characterized by multiple globular hyperfluorescent dilations along branch arteries, extensive “fishnet-like” and cystic capillary dilation, and associated capillary nonperfusion areas. (C) Figure corresponds to case M2 (UWFI, Fig. 1I) and shows type 1 macular telangiectasia. Telangiectatic capillaries are visible in the macular region, with multiple punctate hyperfluorescent spots inferior to the macula and in the temporal periphery, large areas of “fishnet-like” dilated capillaries, and nonperfusion zones. (D, E) Figures correspond to cases L1 and L3, respectively, and show peripheral abnormalities in the fellow eyes of patients with Leber miliary aneurysm. (D) Figure shows tortuous small vessels, telangiectatic capillaries, mild leakage, and microaneurysms in the temporal periphery of the fellow eye. (E) Figure similarly shows tortuous small vessels and telangiectatic capillaries in the fellow-eye periphery. (F, G) correspond to cases M2 and M5, respectively, and show peripheral abnormalities in the affected eyes of patients with type 1 macular telangiectasia, including the nasal periphery in M2 and the temporal periphery in M5. Both images show nonperfusion areas, tortuous peripheral small vessels, telangiectatic capillaries, mild leakage, and a few microaneurysms. UW-FFA = ultra-widefield fluorescein angiography, UWFI = ultra-wide field fundus imaging.

OCT examination revealed varying degrees of macular edema with hyperreflective masses in 14 cases and an epiretinal membrane in 5 cases.

## 4. Discussion

George Coats^[8]^ first described Coats disease in 1908 as an idiopathic retinal vascular disorder characterized mainly by abnormal dilation of retinal capillaries, miliary aneurysms, and intraretinal and/or subretinal yellowish exudation. Later, Leber and Reese described another condition with similar features but without the massive exudation seen in Coats disease, naming it Leber’s miliary aneurysm (LMA). This lesion may eventually progress to Coats disease, and many scholars consider LMA to be an early stage of Coats disease.^[9]^ In 2006, Yannuzzi^[10]^ classified idiopathic macular telangiectasia (MacTel), with MacTel 1 regarded as a form of Coats disease. The imaging manifestations of adult-onset Coats disease differ from those in children. With the development of wide-field fundus imaging systems, observation of such diseases has become more convenient and detailed, especially for peripheral lesions in adult patients, addressing the lack of such data in previous studies.

Our study shows that the extent and morphology of exudative lesions in adult-onset Coats disease patients can be similar to those in children or relatively localized. Typical Coats disease fundus findings were present in 40.0% of cases, indicating that extensive exudation is not uncommon in adult patients. Previous studies have found that myopia and axial elongation may have a protective effect on diabetic retinopathy by reducing intraocular VEGF concentration, among other pathways.^[11-14]^ We closely observed the exudation patterns in patients C1 to C6. Patients C1 and C2 had very extensive exudation, but the main exudative lesions had relatively smooth and clear borders. In patients C3 to C6, the exudative borders were more irregular and indistinct, and fundus photographs of C4, C5, and C6 showed varying degrees of fibrous tissue and hemorrhagic nodules (localized solid elevations reported on ocular B‑scan). Regarding refractive status, C1 and C2 had low myopia (<1 D), whereas C3–C6 had moderate to high myopia (>3 D). It is speculated that moderate‑high myopia may influence the natural course of exudative lesions by affecting retinal metabolism, but due to the small sample size, larger studies are needed for verification.

Over 20 years ago, Shields et al^[7]^ published the largest clinical study on Coats disease (150 cases) using standard fundus photography and fluorescein angiography. Previous studies^[1,2,15]^ have shown that both adult and pediatric Coats disease predominantly affect males and are unilateral. In our study, males accounted for 80.0%, and unilateral involvement for 93.3%, consistent with prior reports. Although Coats disease is generally considered unilateral, recent international studies using UW‑FFA^[16-19]^ have found that 51.7% to 100% of patients (without age stratification) show various pathological changes in the fellow eye, including peripheral vascular tortuosity, capillary dilation, mild leakage, microaneurysms, and non‑perfusion areas. In our observations, apart from case No. 4 who had bilateral involvement, the fellow eyes of other patients showed no abnormalities such as retinal capillary dilation, aneurysms, vascular sheathing, exudation, or hemorrhage on UWFI. However, UW‑FFA revealed abnormalities in 8 patients (53.3%). The presence and severity of fellow‑eye abnormalities did not correlate with the severity of the affected eye. Notably, all patients meeting LMA criteria (L1–L4) had fellow‑eye abnormalities on UW‑FFA. Lai et al^[20]^ reported clinical features of 3 LMA cases, noting no obvious abnormalities in fellow‑eye fundus photographs, but only performed FFA on the affected eye without describing fellow-eye FFA. Although these patients had relatively small but numerous scattered exudative lesions and widespread capillary dilation on UW-FFA, fellow‑eye vascular abnormalities may be related to their unique pathological characteristics.

MacTel 1 is characterized by telangiectasia of the macular capillary network, aneurysmal dilation, and lipid deposition. Over 20 years ago, Yannuzzi et al^[10]^ observed that 2 out of 10 patients examined with conventional FFA had lesions not confined to the macula, with peripheral involvement (though specific details regarding mid‑ or far‑periphery and lesion types were not provided). Considering the limitations of conventional FFA for far‑peripheral observation, our study examined both macular and peripheral vascular changes. All patients meeting MacTel 1 criteria (M1–M5) showed varying degrees of peripheral retinal abnormalities, a finding rarely reported, and only Pollmann,^[21]^ Bohórquez,^[22]^ and Cirafici^[23]^ have reported similar changes in 5 cases. Among these 10 patients, the most notable peripheral finding was non‑perfusion areas, further supporting that MacTel 1 is a subtype of Coats disease and reflecting a peripheral retinal ischemic‑hypoxic state. This provides new directions for guiding treatment and exploring the pathogenesis of this condition.

Adult-onset Coats disease is relatively rare in clinical practice, and many other retinal vascular abnormalities may present with Coats-like manifestations such as retinal telangiectasia, massive exudation, and even miliary aneurysms. It must be differentiated from exudative age-related macular degeneration, chronic retinal vein occlusion, chronic posterior uveitis, chronic retinal vasculitis, and retinal macroaneurysms. Comprehensive mastery of the clinical characteristics of adult-onset Coats disease and various diseases causing retinal telangiectasia, detailed understanding of the medical history, and thorough analysis of the condition – particularly in combination with UW-FFA findings – are crucial for correct diagnosis. However, conditions such as Parry-Romberg syndrome, which involve abnormalities in other parts of the body while the fundus manifestations may be entirely similar, require confirmation with cranial CT.^[24]^ Diagnosis of bilateral adult-onset Coats disease should be approached with even greater caution. In addition to differentiation from extensively exudative diabetic retinopathy, it is also necessary to exclude Coats-like retinopathies associated with inherited metabolic diseases and syndromes.^[25]^ For example, in RP combined with Coats-like retinopathy, RP is usually diagnosed first, followed by the development of Coats-like changes. It usually occurs bilaterally with no gender predilection. FSHD typically manifests around age 20, but most patients exhibit significant facial and shoulder girdle muscle weakness and atrophy. Moreover, the median age at diagnosis of Coats syndrome in such cases is 10 years,^[26]^ making differentiation relatively straightforward.

OCT, as a convenient non‑invasive examination, can detect secondary changes in adult-onset Coats disease such as exudative lesions, subretinal fluid, macular edema, epiretinal membrane, and fibrous nodules. It also aids in monitoring changes before and after treatment. Dense exudation in the macula, fibrous nodules, and loss or disruption of the ellipsoid zone on OCT suggest poorer baseline and prognostic visual acuity.^[27,28]^ Our observation found that patients with dense hyper‑reflective particles in the foveal center on OCT, except for 1 case with BCVA of 0.12, all had BCVA below 0.05. However, due to the retrospective nature, small sample size, varying treatment interventions, and inconsistent follow‑up, we cannot analyze patient prognosis.

Overall, We have preliminarily summarized some imaging features of adult-onset Coats disease, particularly new findings on UW‑FFA. Further studies with larger sample sizes are needed to explore more characteristic changes.

## Author contributions

**Conceptualization:** Guangwei Yu, Yanhui Han.

**Data curation:** Fanchao Meng, Feng Chen.

**Methodology:** Haohao Lu.

**Supervision:** Feng Chen, Yanhui Han.

**Visualization:** Haohao Lu.

**Writing – original draft:** Guangwei Yu.

**Writing – review & editing:** Guangwei Yu.
